# Supplementary material for: Emergence of orbital angular moment at van Hove singularity in graphene/h-BN moiré superlattice
Source: Nat Commun. 2020 Oct 23;11:5380. doi: 10.1038/s41467-020-19043-x (PMC7584618; doi:10.1038/s41467-020-19043-x)
Supplement: Supplementary file 1 — Supplementary Information [file 41467_2020_19043_MOESM1_ESM.pdf]

—Supplementary information—

Emergence of orbital angular moment at van Hove singularity in graphene/*h*-BN moiré superlattice

Rai Moriya[1,*], Kei Kinoshita[1], J. A. Crosse[2], Kenji Watanabe[3], Takashi Taniguchi[4,1], Satoru Masubuchi[1], Pilkyung Moon[2,5], Mikito Koshino[6], and Tomoki Machida[1,*]

[1] *Institute of Industrial Science, University of Tokyo, 4-6-1 Komaba, Meguro, Tokyo 153-8505, Japan*

[2] *New York University Shanghai and NYU-ECNU Institute of Physics at NYU Shanghai, Shanghai, China*

[3] *Research Center for Functional Materials, National Institute for Materials Science, 1-1 Namiki, Tsukuba 305-0044, Japan*

[4] *International Center for Materials Nanoarchitectonics, National Institute for Materials Science, 1-1 Namiki, Tsukuba 305-0044, Japan*

[5] *State Key Laboratory of Precision Spectroscopy, East China Normal University, Shanghai 200062, China*

[6] *Department of Physics, Osaka University, Toyonaka, Osaka 560-0043, Japan*

*E-mail: moriyar@iis.u-tokyo.ac.jp; tmachida@iis.u-tokyo.ac.jp

Supplementary Note 1

Calculating the band structure of aligned graphene/*h*-BN moiré superlattice under inversion symmetric and inversion asymmetric conditions. We used the effective continuum model for calculating the band structure of the graphene/*h*-BN system [1]. The effective reduced Hamiltonian of the system near the $\mathbf{K}_\xi$ point is written as

$$H_{\text{G-hBN}} = H_{\text{G}} + U^{\dagger}(-H_{\text{hBN}})^{-1}U$$
$$\equiv H_{\text{G}} + V_{\text{hBN}}, \qquad (1)$$

where $\xi = \pm 1$ for the K and K′ valleys, H_{G} is the Hamiltonian of monolayer graphene near $\mathbf{K}_\xi$, and V_{hBN} is written as

$$V_{\text{hBN}} = V_0\begin{pmatrix}1 & 0\\ 0 & 1\end{pmatrix} + \left\{V_1 e^{\mathrm{i}\xi\psi}\left[\begin{pmatrix}1 & \omega^{-\xi}\\ 1 & \omega^{-\xi}\end{pmatrix} e^{\mathrm{i}\xi\mathbf{G}_1^{\mathrm{M}}\cdot\mathbf{r}} + \begin{pmatrix}1 & \omega^{\xi}\\ \omega^{\xi} & \omega^{-\xi}\end{pmatrix} e^{\mathrm{i}\xi\mathbf{G}_2^{\mathrm{M}}\cdot\mathbf{r}} + \begin{pmatrix}1 & 1\\ \omega^{-\xi} & \omega^{-\xi}\end{pmatrix} e^{-\mathrm{i}\xi(\mathbf{G}_1^{\mathrm{M}}+\boldsymbol{G}_2^{M})\cdot\mathbf{r}}\right] + \text{H.c.}\right\} \qquad (2)$$

where

$$V_0 = -3u_0^2\left(\frac{1}{V_{\text{N}}} + \frac{1}{V_{\text{B}}}\right), \qquad (3)$$

$$V_1 e^{i\psi} = -u_0^2\left(\frac{1}{V_{\text{N}}} + \omega\frac{1}{V_{\text{B}}}\right). \qquad (4)$$

We set the parameters $u_0 \approx 0.152$ eV, $V_0 \approx 0.0289$ eV and $V_1 \approx 0.0210$ eV to the same values as the previous publication [1]. For the inversion symmetric model, we set $\psi = \pi/3$ radian so that the system satisfies the inversion symmetric condition. For the inversion asymmetric model, we set $\psi \approx -0.29$ radian. Results are presented in Supplementary Figure 1. The inversion asymmetric calculation demonstrated good coincidence with the experimentally observed behavior of graphene/*h*-BN moiré superlattice [1-4].

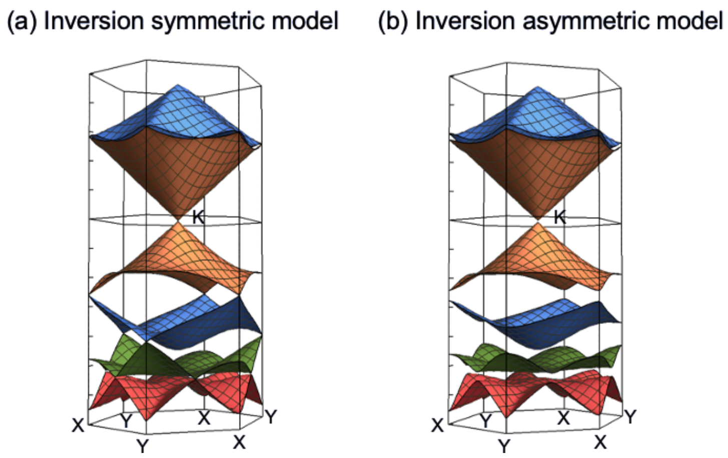

Supplementary Figure 1: Calculated band structure of the graphene/*h*-BN moiré superlattice

(a,b) Band structure of the graphene/*h*-BN moiré superlattice with $\theta = 0°$ at K-point calculated using an effective continuum model. Calculation is performed under an (a) inversion symmetric model and an (b) inversion asymmetric model.

Supplementary Note 2

Comparison of orbital magnetic moment among different bands in graphene/*h*-BN moiré superlattice. In Supplementary Figure 2 (e,f,g), we show the detailed comparison between the calculated **m**(**k**) values obtained from different bands. In particular, a data sets of calculated **m**(**k**) versus **k** are presented. The distribution of **m**(**k**) in the hole-side 1st band is shown in panel (b). The line profile of **m**(**k**) along the line between the Y-point and X-point is shown in panel (e). The Y-point and X-point correspond to the 1st vHS(h) and hole-side secondary Dirac point [SDP(h)], respectively. We obtained **m**(**k**) values of 6.7μ_B [1st vHS(h)] and 134.6μ_B [SDP(h)], respectively. Similarly, the line profile of the second band shown in panel (c) is presented in panel (f). Here, the Y-point and X-point of the 2nd band correspond to the 2nd vHS(h) and SDP(h), respectively. Then we obtained **m**(**k**) values of 66.4μ_B [2nd vHS(h)] and 164.1μ_B [SDP(h)], respectively. Next, the third band in panel (d) has a rather complex band structure. The line profile between the Y- and X-point is shown in panel (g). Here, the Y-point of the 3rd band corresponds to TDP(h) and exhibits an **m**(**k**) value of 42.0μ_B. From these comparisons, a large **m**(**k**) value is found at gapped DPs [main DP, SDP(h), and TDP(h)] and the 2nd vHS(h). The **m**(**k**) value is small for the 1st vHS(h). Therefore, among vHSs, the 2nd vHS(h) has an exceptionally large **m**(**k**) value compared to the other vHSs. The obtained **m**(**k**) = 66.4 μ_B corresponds to the valley g-factor of $g = 2\mathbf{m}(\mathbf{k}) = 132.8$. We note that this value showed good coincident with the g-factor obtained from the energy splitting calculation between the K and K′ valleys at the 2nd vHS under the magnetic field (see Supplementary Note 7).

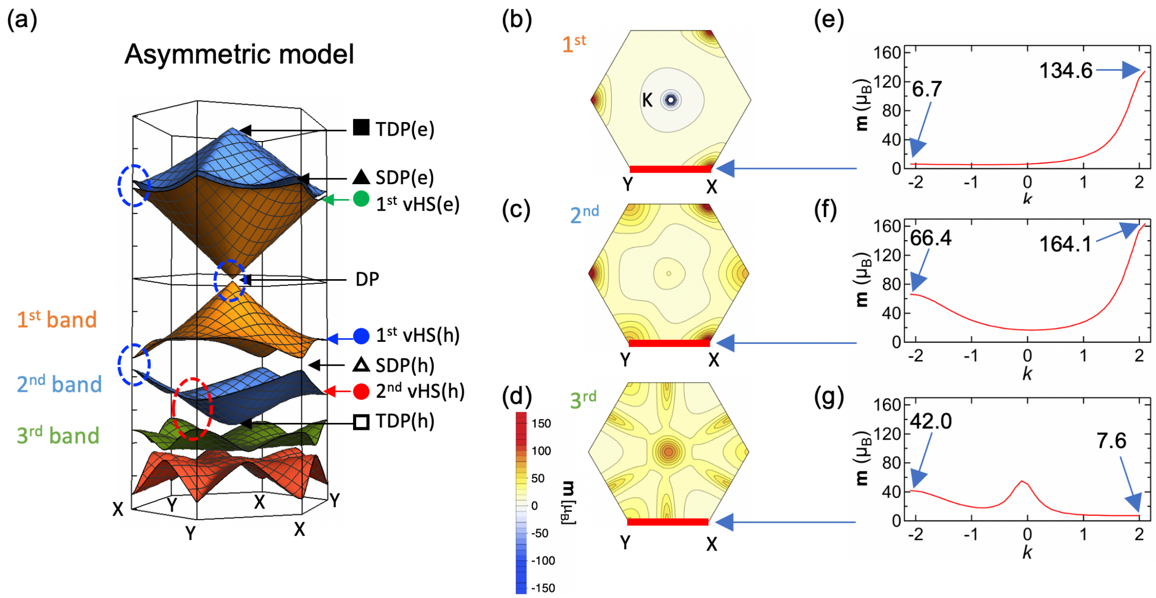

Supplementary Figure 2: Calculated orbital angular moment of the graphene/*h*-BN moiré superlattice

(a) Band structure of the graphene/*h*-BN Moiré superlattice with $\theta = 0°$ at K-point calculated using an effective continuum model. Calculation is performed under an inversion asymmetric model. (b,c,d) Calculated orbital angular moment $\mathbf{m}(\mathbf{k})$ for hole-side (b) first band, (c) second band, and (d) third band. (e,f,g) Line profile of $\mathbf{m}(\mathbf{k})$ vs. $\mathbf{k}$ between the Y-point and X-point for (e) first band, (f) second band, and (g) third band.

Supplementary Note 3

Effect of creating trench in *h*-BN for reducing capacitive coupling between graphene flakes. The main purpose of the making cut in the *h*-BN in our device is to reduce capacitive coupling between the two graphene flakes during the thermoelectric measurement as we illustrated in Supplementary Figure 3. Due to the capacitive coupling, we saw a crosstalk between the graphene flakes during measurement particularly for the operation of the graphite gate. Several optimizations were performed in our device structure for reducing the crosstalk. The more distance between graphite gate and other graphene, as well as an introduction of cutting in the *h*-BN between graphene flakes helped to reduce electrical crosstalk. The drawback of having longer distance between two graphene flakes is reducing heat transfer efficiency from heater graphene to another graphene. More heater power is required to observe the thermoelectric signal in the device having longer separation. The structure shown in our manuscript was optimized for minimizing capacitive coupling and at the same time having reasonable heating efficiency. The presence of cutting in the *h*-BN seemed to be a small influence for heat transfer between the graphene flakes. So, we think that the effect of cutting *h*-BN is mainly for reducing electrical noise during measurement.

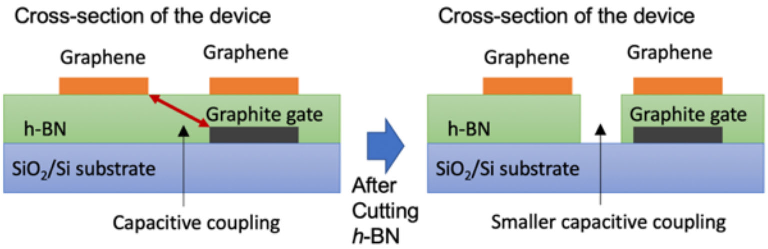

Supplementary Figure 3: Effect of trench in *h*-BN in the device

Illustration of the influence of the trench in the *h*-BN between two graphene flakes.

Supplementary Note 4

Evaluation of contact resistance of graphene heater. The two-terminal resistance of the heater graphene under the low magnetic field region B = 0.0, 1.0, and 2.0 T is presented in Supplementary Figure 4(a). First, we extract the contact resistance contribution of the device by the following method. At the highest carrier density (n_R = $\pm 2.37\times10^{12}$ cm^{-2}), resistance is saturated at the lowest value. As a rough estimation of contact resistance, we assumed that the two-terminal resistance at these highest carrier densities is dominated by the two metal/graphene contact resistance. Then we obtained ~5.0 kΩ and ~3.4 kΩ for the hole-doped (n_R = -2.37×10^{12} cm^{-2}) and electron-doped (n_R = $+2.37\times10^{12}$ cm^{-2}) sides. Next contact resistance can be also estimated from the deviation of two-terminal resistance from the quantum Hall resistance value at the high magnetic field as shown in Supplementary Figure 4(b). The quantum Hall resistance at filling factor v = ±2 and 6 is 12.906 kΩ and 4.3021 kΩ, respectively (these values are indicated by the dashed line in the figure). The difference between these values and measured two-terminal resistance can be attributed to the contact resistance contribution. Obtained values at v = ±2 and 6 are plotted in Supplementary Figure 4(c) as solid red squares together with the contact resistance extracted by the first method (solid black squares). Both measurements provide similar values for a contact resistance of ~5 kΩ and ~3 kΩ for the hole and electron-doped sides, respectively. During the thermopower measurement when applying the power of 1 mW to the heater graphene, we adjusted the gate voltage to tune the carrier density of the heater graphene to be at v = -6; so the two-terminal device resistance is maintained around ~10 kΩ [Supplementary Figure 4(b)]. Note that the two-terminal device resistance at DP is also ~10 kΩ. Therefore, the device resistance of heater graphene

(contact resistance + graphene's channel resistance) is always maintained at ~10 kΩ from no magnetic field to the high magnetic field. In this way, we achieved constant resistance of the heater graphene throughout the measurement. Since the dimension of the heater graphene channel is 9.4 μm (length) and 3.4 μm (width), the sheet resistance of the graphene channel under above-mentioned condition is ~1.5 kΩ/□. From these comparisons, the contact resistance and channel resistance are of a similar order. Both contact resistance and channel resistance are much higher than the resistance of the Au/Cr electrode and wiring, making us believe that at least heat generation is localized around the heater graphene device (heater graphene channel and interface between heater graphene and metal electrode).

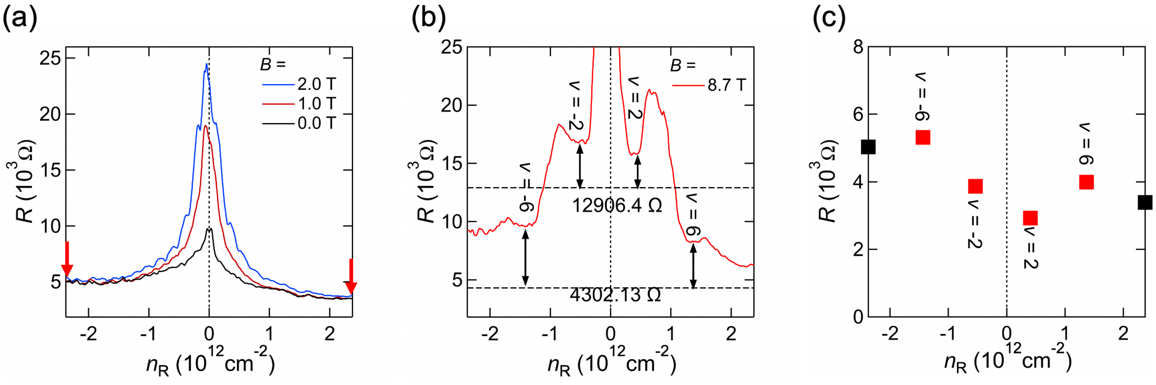

Supplementary Figure 4: Transport properties of heater graphene

(a) Two-terminal resistance R of a heater graphene as a function of carrier density n_R measured under different magnetic field B measured at 2.0 K. (b) R vs. n_R at B = 8.7 T. Quantum Hall plateaus of filling factor ν = -6, -2, +2, and +6 are clearly visible. (c) Total contact resistance contribution of the Au/Cr/graphene junction (solid red square) determined for different n_R values. The contact resistance contribution determined from the saturation of panel (a) is plotted as a solid black square.

Supplementary Note 5

Magnetothermoelectric signal from nonaligned graphene/*h*-BN device. A magnetothermopower signal was calibrated using a measurement geometry, as illustrated in Supplementary Figure 5(a). Here, we used aligned graphene, depicted on the left-side, as a heater by passing a current thorough it and detected the thermopower generated on the nonaligned grapheme, depicted in the right-side. A constant power was applied to the graphene on the left using a current source while its carrier density was maintained at filling factor $\nu = +6$ such that $n_L = 6eB/h$, where e depicts electron charge and h the Planck constant. The two-terminal resistance R of the graphene on the right is presented in Supplementary Figure 5(b), measured at 2.0 K. Supplementary Figure 5(c) shows the detected thermopower V_{ind} for the graphene on the right under the application of constant power P = 1 mW to the graphene on the left as a function of n_R at different magnetic fields B. At B = 0 T, the V_{ind} signal is negligibly small but it starts to develop with an increase of B. The signal at DP ($n_R \sim 0$) increases with B, and its sign changes with the direction of the magnetic field, which is reminiscent of the Nernst effect [5-7]. The image plot of V_{ind} under the sweep of B and n_R at 2.0 K is shown in Supplementary Figure 5(d); this plot shows the development of Landau levels upon application of the magnetic field. Thus, the thermoelectric signal observed in this device originated from DP and its Landau quantization. The Nernst signal at maximum magnetic field of 8.6 T and measurement temperature of 2.0 K is approximately 100 μV (from Supplementary Figure 5(c) and 5(d)). According to the literature, the Nernst coefficient of graphene at similar temperature and magnetic field values is approximately 10–20 μV/K [5,8]. From these comparisons, the estimated temperature rise of the graphene on the right due to the heat transfer from the

graphene on the left is about 5−10 K. Since the measurement temperature is 2.0 K, we can estimate the maximum temperature rise of the graphene to be 7 ~ 12 K.

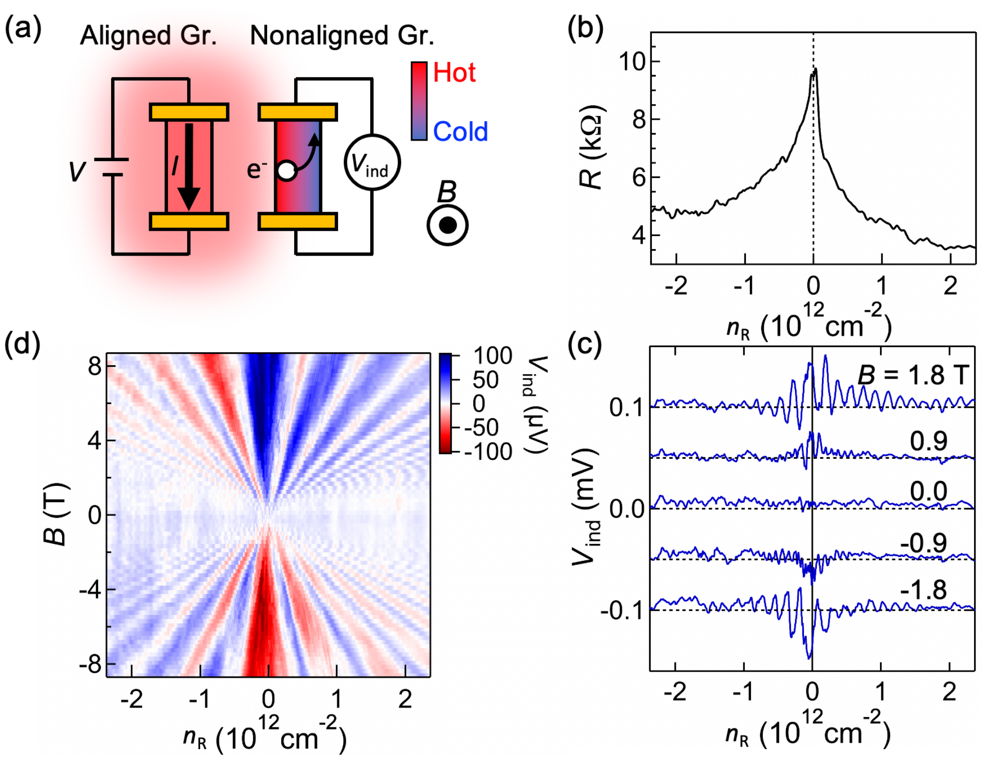

Supplementary Figure 5: Magnetothermoelectric signal from nonaligned graphene/*h*-BN device

(a) Schematic illustration of thermoelectric voltage detection in a parallel graphene device. (b) Two-terminal resistance R of the nonaligned graphene on the right as a function of its carrier density n_R at 2.0 K. (c) Thermoelectric voltage V_{ind} generated in the nonaligned graphene as a function of its carrier density n_R, measured at a different out-of-plane magnetic field $B = \pm 1.8$, 0.9, and 0 T. A constant power P of 1 mW is applied to the aligned graphene on the left. (d) V_{ind} as a function of n_R and B.

Supplementary Note 6

Difference in the magnetothermoelectric response between DPs and vHSs.

Here, we discuss the difference in the magnetothermoelectric signal between DPs and vHSs. As we see from Supplementary Figure 5, the Nernst effect in pristine graphene exhibits sign reversal between +B and -B. This is general behavior of the Nernst effect not only for graphene but also for other material; because of the reversal of the Lorentz force between different B directions. The Nernst voltage V_{NR} in the geometry shown in Supplementary Figure 6 can be expressed as follows [5,7,9];

$$V_{\mathrm{NR}} \propto (\sigma^{-1})_{\mathrm{xx}} \left(\frac{\partial \sigma}{\partial E_{\mathrm{F}}}\right)_{\mathrm{xy}} + (\sigma^{-1})_{\mathrm{xy}} \left(\frac{\partial \sigma}{\partial E_{\mathrm{F}}}\right)_{\mathrm{yy}}, \qquad (5)$$

where σ depicts the conductivity tensor and E_{F} the Fermi energy. The sign of V_{NR} changes under the reversal of B due to the sign reversal of σ_{xy}. In addition to this, according to Supplementary Equation 5, the sign of V_{NR} should be opposite between the Dirac point (DP) and vHS. This is because majority carrier changes from electron to hole across DP, from the higher energy side to lower energy side. In contrast, the majority carrier changes from hole (higher energy) to electron (lower energy) across the vHS. Thus, the slope of σ_{xy} vs. E_{F} is opposite between the DPs and vHSs. Consequently, the sign of the Nernst signal

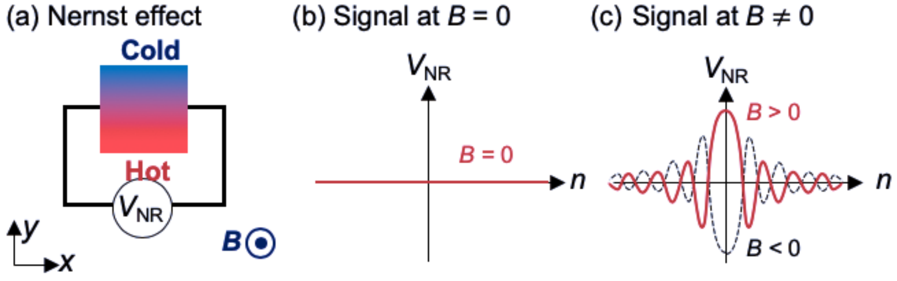

Supplementary Figure 6: Schematic illustration of the Nernst effect

(a) Schematic structure of a device exhibiting the Nernst effect. (b,c) Graphene's carrier density n dependence on the Nernst signal. Illustrations are presented for both (b) zero magnetic field and (c) high magnetic fields.

is opposite between the DP (as well as LLs originated from the DP) and vHS points. Therefore, B dependence of the Nernst signal can be used to distinguish whether the signal originated from the DP or the vHS. An example of this is shown in the Supplementary Figure 7. We plot the Nernst voltage V_{ind} as a function of B for four different DPs. These

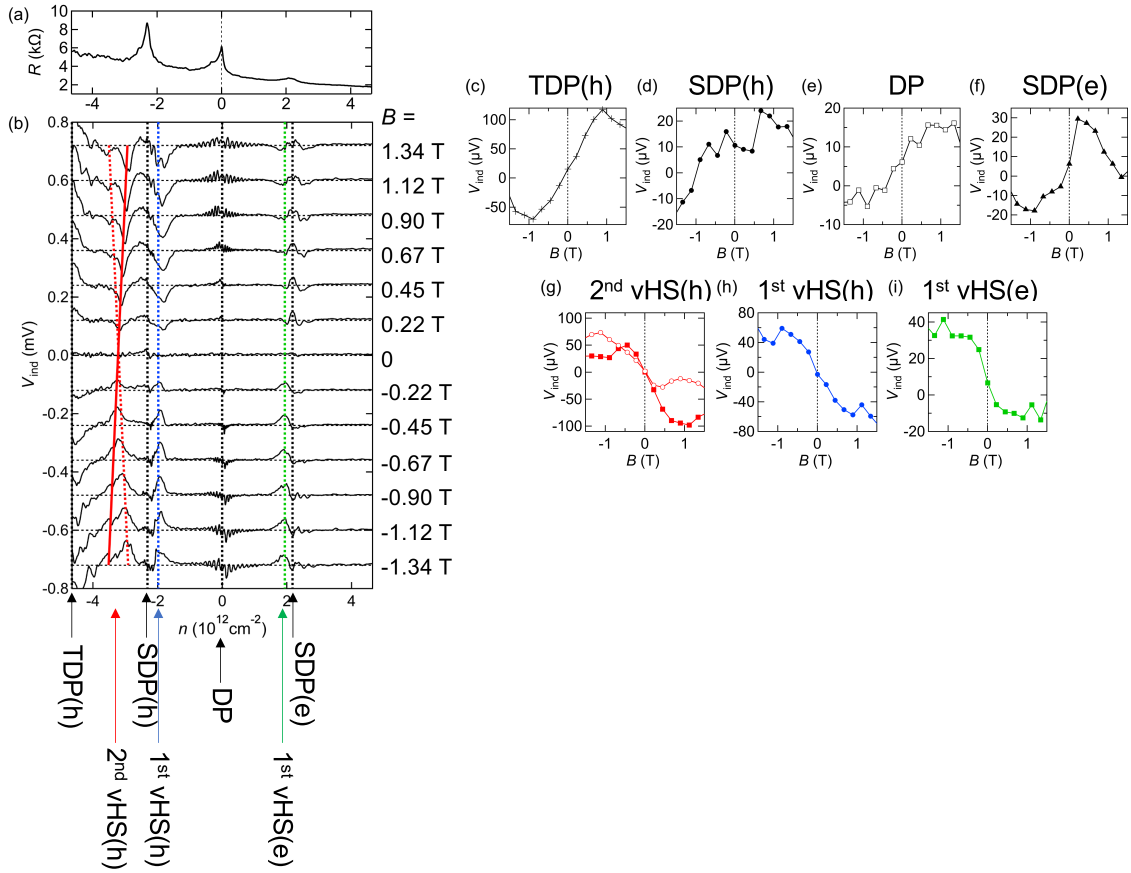

Supplementary Figure 7: Magnetothermoelectric signal of DPs and vHSs

(a) Two-terminal resistance R of the graphene on the left as a function of its carrier density n_{L} at 2.0 K. (b) V_{ind} as a function of the carrier density n_{L} measured at a different out-of-plane magnetic field B obtained from the graphene/h-BN moiré superlattice device at 2.0 K. The dotted black lines depict the carrier density of the DP, SDP(e), and SDP(h). The red, blue, and green lines depict the 2nd vHS(h), 1st vHS(h), and 1st vHS(e), respectively. Traces are offset for clarity and the offset is depicted by black dashed lines. (c,d,e,f) V_{ind} as a function of the B for four DPs of (c) TDP(h), (d) SDP(h), (e) DP, and (f) SDP(e). (g,h,i) V_{ind} as a function of the B for three vHSs of (g) 2nd vHS(h), (h) 1st vHS(h), and (i) 1st vHS(e).

are the main Dirac point (DP), electron- and hole-side secondary Dirac point (SDP), and hole-side third (or tertiary) Dirac point (TDP). The signal from these points tends to increase with B while the signals from all the vHSs decrease with B. Thus, there is an apparent difference in the sign of the V_{ind} signals. This information is used to separate the signal from the DPs and vHSs. With this information, the bottom of the dip or the top of the peak of V_{ind} signal are taken as a position of the vHSs and the plot shown in Fig. 3(b) is constructed. All the assignment of Landau levels and vHSs are presented in Supplementary Figure 8.

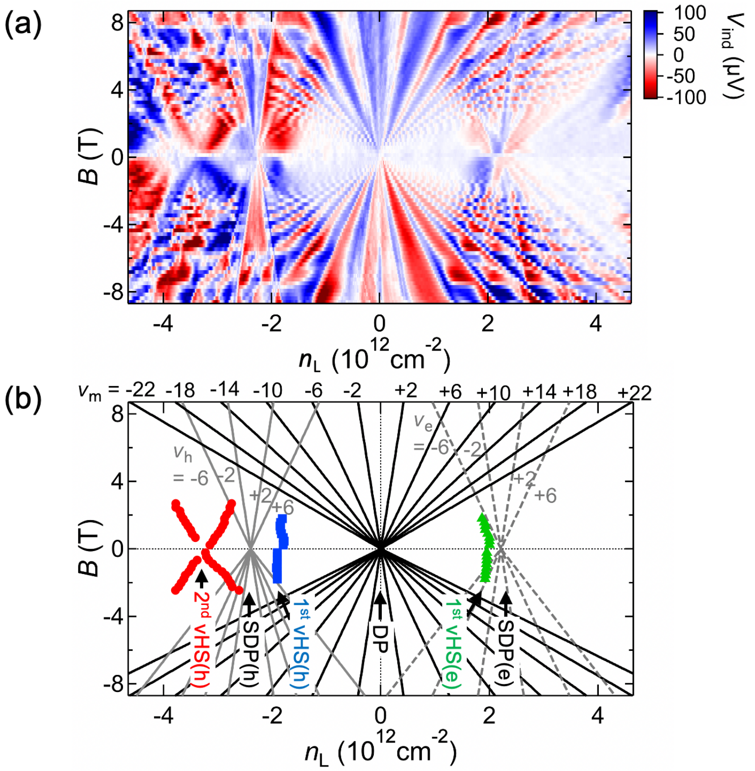

Supplementary Figure 8: High magnetic field magnetothermoelectric signal of the graphene/*h*-BN moiré superlattice

(a) V_{ind} as a function of n_L and B. (b) Landau level fan diagram for main DP, hole-side secondary DP [SDP(h)], and electron-side secondary DP [SDP(e)] are depicted by solid black, solid gray, and dashed gray lines, respectively. The Landau level filling factors of DP, SDP(h), and SDP(e) are indicated as ν_m, ν_h, and ν_e, respectively. Peak positions of V_{ind} at each vHS obtained from (a) plotted as a solid red circle [second vHS(h)], solid blue circle [first vHS(h)], and solid green circle [first vHS(e)].

Supplementary Note 7

Calculated density of states versus energy in graphene/*h*-BN moiré superlattice. In Supplementary Figure 9, we present a calculated DOS as a function of energy E and magnetic field B. Here, we plotted the total DOS, including the DOS for both the K and K′ valleys [Supplementary Figure 9(a)], DOS for the K valley [Supplementary Figure 9(b)], and DOS for the K′ valley [Supplementary Figure 9(c)], separately. The DOS vs. E for several B values are plotted in Supplementary Figure 9(d). The 2nd vHS(h) indicated by a filled red circle in the trace for $B = 0$ shows splitting upon the increse of B. The each of split DOSs are depicted by filled and open red circles. The peak energy of the DOSs is plotted in with respect to B in Supplementary Figure 9(e). Then, the splitting energy ΔE between the two valleys versus B is shown in Supplementary Figure 9(f). The valley g-factor g is calculated from the relation, $g = \Delta E/\mu_B B$ where μ_B denotes the Bohr magneton. From this analysis, we obtained $g \sim 116$. From the semiclassical calculation of $\mathbf{m}(\mathbf{k})$ discussed in Supplementary Note 2, we obtained $\mathbf{m}(\mathbf{k}) \sim 66\ \mu_B$ at the 2nd vHS(h); this corresponds to the g-factor of $g = 2\ \mathbf{m}(\mathbf{k}) \sim 132$. Therefore, these two calculated values are reasonably consistent each other.

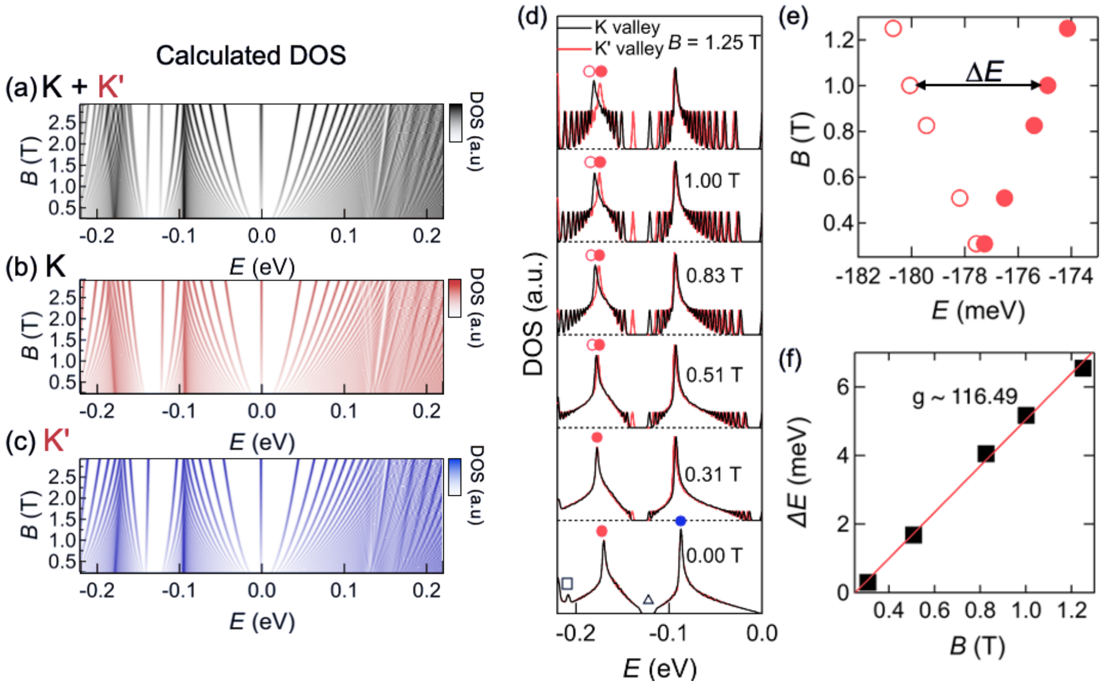

Supplementary Figure 9: Calculation of K and K′ valley splitting in the perpendicular magnetic field

(a,b,c) Calculated DOS as a function of the energy E and B under the condition of (a) total DOS including both the K and K′ valleys, (b) DOS of the K valley, and (c) DOS of the K′ valley. (d) DOS with respect to the energy E calculated at different magnetic field values. Traces are offset for clarity and the offset is depicted by black dashed lines. (e) The peak positions of DOS for 2nd vHS(h) versus different magnetic field B. (f) The energy splitting ΔE versus B.

Supplementary Note 8

The origin of large orbital moment at 2nd hole-side vHS. The semiclassical model for describing orbital magnetic moments is given by the following expression [10,11];

$$\mathbf{m}(\mathbf{k}) = -\mathrm{i}\frac{\mathrm{e}}{2\hbar}\langle\boldsymbol{\nabla}_{\mathrm{k}}u| \times [H(\boldsymbol{k}) - \varepsilon^{0}(\boldsymbol{k})]|\boldsymbol{\nabla}_{\mathrm{k}}u\rangle, \qquad (6)$$

where, $|u(\mathbf{k})\rangle$ is the periodic part of the Bloch function, $H(\mathbf{k})$ is the Bloch Hamiltonian, and $\varepsilon^0(\mathbf{k})$ is the dispersion of band. This equation can be rewritten into the following form [12-14];

$$\mathbf{m}_n(\mathbf{k}) = -\mathrm{i}\frac{\mathrm{e}}{2\hbar}\sum_{j\neq n}\frac{\mathbf{P}_{n,j}(\mathbf{k})\times\mathbf{P}_{j,\mathrm{n}}(\mathbf{k})}{\varepsilon_j^0(\mathbf{k})-\varepsilon_n^0(\mathbf{k})}, \qquad (7)$$

Here, $\mathbf{P}_{n,j}(\mathbf{k}) \equiv \langle u_{n,\mathbf{k}}|\widehat{\mathbf{p}}|u_{j,\mathbf{k}}\rangle$ is the interband matrix element of the canonical momentum operator $\widehat{\mathbf{p}}$. $\varepsilon_n^0(\mathbf{k})$ is the dispersion of nth band and $\varepsilon_n(\mathbf{k}) = \varepsilon_n^0(\mathbf{k}) - \mathbf{m}_n(\mathbf{k})\cdot\mathbf{B}$ is the electron energy. To obtain a finite orbital moment, the both numerator and denominator need to be nonzero in Supplementary Equation 7. Since the denominator of Supplementary Equation 7 corresponds to the energy separation to neighboring bands, it is mostly nonzero everywhere in the band except the point of contact between the bands such as the Dirac point (zero-gap Dirac point).

Then, the numerator, the interband matrix element, is crucial for obtaining large $\mathbf{m}(\mathbf{k})$. In an inversion symmetric model, this value is finite only at the point where two bands make contact as indicated by red arrows in Supplementary Figure 10(a). The interband matrix elements at this point will be conserved to be finite even after the gap opening under to the introduction of inversion asymmetry. Below, we present a few examples of these results. Point A in Supplementary Figure 10(a) is the most interesting as this point is the point at which the band meet in the symmetric model, making the numerator of Supplementary Equation 7 finite. In other words, this point satisfies the

criteria of the finite interband matrix element. In the asymmetric model [Supplementary Figure 10(b)], a large gap is opened at this point which generate the 2nd vHS(h) point in the second band and TDP(h) in the third band. Since there is a finite gap between the neighboring band point, both 2nd vHS(h) point in the second band and TDP(h) in the third band exhibit a large orbital moment.

In comparison, Point B is not the point of contact between the bands, but a vHS of the 1st band in the symmetric model. Thus the numerator, the interband matrix element, is zero. In the asymmetric model, this point also the vHS of the 1st band. Introduction of inversion asymmetry slightly modified the band structure to generate finite orbital moment $\mathbf{m}(\mathbf{k})$; however, since the numerator, the interband matrix, maintains a small value even after the introduction of inversion asymmetry, the obtained $\mathbf{m}(\mathbf{k})$ is small at Point B [see Supplementary Figure 2].

Other points of contact between the bands in the symmetric model (Point C, D, and E) will be gaped in the inversion asymmetric model and become main- and secondary-DP; these points have a large orbital moment as shown in Supplementary Figure 2. Overall, the point of contact between the bands in the symmetric model always shows large orbital moment when this point is gapped under the inversion asymmetric model. This explains our experimental observation of a large orbital moment at the 2nd vHS(h).

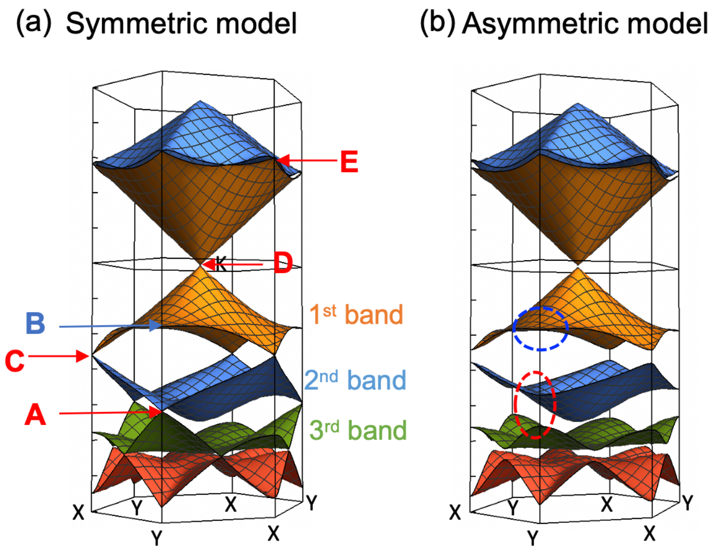

Supplementary Figure 10: Calculated band structure of the graphene/*h*-BN moiré superlattice

(a,b) Band structure of the graphene/*h*-BN moiré superlattice with $\theta = 0°$ at K-point calculated using an effective continuum model. Calculation is performed under an (a) inversion symmetric model and an (b) inversion asymmetric model.

Supplementary references

1 Moon, P. & Koshino, M. Electronic properties of graphene/hexagonal-boron-nitride moiré superlattice. *Phys. Rev. B* **90**, 155406 (2014).

2 Hunt, B. *et al.* Massive Dirac fermions and Hofstadter butterfly in a van der Waals heterostructure. *Science* **340**, 1427-1430 (2013).

3 Dean, C. R. *et al.* Hofstadter's butterfly and the fractal quantum Hall effect in moiré superlattices. *Nature* **497**, 598–602 (2013).

4 Ponomarenko, L. A. *et al.* Cloning of Dirac fermions in graphene superlattices. *Nature* **497**, 594–597 (2013).

5 Zuev, Y. M., Chang, W. & Kim, P. Thermoelectric and magnetothermoelectric transport measurements of graphene. *Phys. Rev. Lett.* **102**, 096807 (2009).

6 Checkelsky, J. G. & Ong, N. P. Thermopower and Nernst effect in graphene in a magnetic field. *Phys. Rev. B* **80**, 081413 (2009).

7 Kinoshita, K. *et al.* Photo-Nernst detection of cyclotron resonance in partially irradiated graphene. *Appl. Phys. Lett.* **115**, 153102 (2019).

8 Wei, P., Bao, W., Pu, Y., Lau, C. N. & Shi, J. Anomalous Thermoelectric Transport of Dirac Particles in Graphene. *Phys. Rev. Lett.* **102**, 166808 (2009).

9 Cao, H. *et al.* Photo-Nernst current in graphene. *Nat. Phys.* **12**, 236–239 (2016).

10 Xiao, D., Chang, M.-C. & Niu, Q. Berry phase effects on electronic properties. *Rev. Mod. Phys.* **82**, 1959–2007 (2010).

11 Xiao, D., Yao, W. & Niu, Q. Valley-contrasting physics in graphene: Magnetic moment and topological transport. *Phys. Rev. Lett.* **99**, 236809 (2007).

12 Chang, M.-C. & Niu, Q. Berry phase, hyperorbits, and the Hofstadter spectrum: Semiclassical dynamics in magnetic Bloch bands. *Phys. Rev. B* **53**, 7010-7023 (1996).

13 Yao, W., Xiao, D. & Niu, Q. Valley-dependent optoelectronics from inversion symmetry breaking. *Phys. Rev. B* **77**, 235406 (2008).

14 Xu, X., Yao, W., Xiao, D. & Heinz, T. F. Spin and pseudospins in layered transition metal dichalcogenides. *Nat. Phys.* **10**, 343–350 (2014).
